# Notes and News

**Published:** 1904-04-16

**Authors:** 


### Notes and News.

The Queen continues to take a great interest in the Finsen Light cure, which she succeeded in installing at the London Hospital some years ago. Last Saturday she visited the Finsen Light Cure Institute in Copenhagen, accompanied by the King. Their Majesties went over the whole building, and conversed with the patients. They also visited Dr. Finsen, who was indisposed.

No more white victims of the Plague are reported from the Transvaal, where there have been upwards of 70 cases altogether.

At the recent annual meeting of the Royal Victoria Hospital, Belfast, great satisfaction was expressed by several speakers at the results of the working of the Plenum system of ventilation at that hospital.

The late Miss Frances Power Cobbe not only provided in her will that means should be taken to prevent any possibility of burial whilst alive, but she also left directions of a sanitary nature to ensure an "earth to earth" interment by the use of a coffin of the most perishable materials.

An inquest held on the body of a child at Wandsworth reveals an extraordinary state of mismanagement at the Bolingbroke Hospital. It is reported that the child was taken to the out-patients' department of the hospital suffering from severe burns. According to the evidence, the child was only once seen by the house surgeon, although it was admitted that the case was one which would have been admitted as an in-patient if there had been a vacant bed. The after-treatment was left entirely to the nurse in the out-patients' department, who could take no action further than dressing the wounds according to directions received. Eventually the child died, the cause of death being certified to be heart failure from incipient pneumonia. The coroner, in summing up, condemned in strong terms the manner in which the child had been treated, and said revision of the methods adopted at the Bolingbroke Hospital was greatly needed. The jury concurred in the coroner's remarks.

Thirty cases of plague are reported from Antofagasta, in Chile.

It is reported that nearly the whole of the Hampshire Regiment, stationed at Aden, have been seized with malarial fever.

Last week, in referring to the Victoria Hospital for Children, Chelsea, we printed an error in describing the accommodation. The new building contains six wards on three floors, with an isolation department at the top of the building.

A strange case has occurred recently at Southampton, where a man was sued for affixing a false signature to a death certificate and claiming a medical degree not his own. On examination it appeared that the name and title adopted by the signatory did not belong to him at all, but as he actually held a doctor's degree of Heidelberg under another name, his counsel pleads that the irregularity shall be lightly dealt with. He has been committed for trial.

The London County Council have ordered that cases of chicken-pox shall be notifiable during a period of four months, owing to the increase in the number of small-pox cases in London and the difficulty of distinguishing between the two diseases. They have further announced that Mr. S. Bingham, formerly Medical Superintendent of one of the small-pox hospitals of the Metropolitan Asylums Board, and Mr. W. McC. Wanklyn, late Small-pox Referee to the Metropolitan Asylums Board and Medical Superintendent of the River Ambulance Service, have been retained by the London County Council, until August 7th, 1904, to assist in the diagnosis of doubtful cases of small-pox. Any medical practitioner desiring to avail himself of the services of one of these gentlemen should apply to the Public Health Department of the London County Council (Telephone No. 2,102 Gerrard). Letters should be addressed, Medical Consultant, Public Health Department, 8 St. Martin's Place, Trafalgar Square, W.C.

The Lord Mayor of Birmingham, speaking at the annual meeting of the Hockley Provident Dispensary, made some admirable remarks, displaying much intelligent observation and common sense. He deprecated the ticket system on the ground that time was wasted and ailments exaggerated, and for several other reasons, one being that the effect was to pauperise. He advocated a payment according to means by all patients except the very poorest. He thought if this custom were adopted that a far greater number would join provident dispensaries than wait for hours in the out-patient departments of the hospitals. Speaking before the meeting of a provident dispensary, the Mayor's remarks were not likely to meet with objections. But there are many hospitals where such sentiments would not be popular. A crowded out-patients' department is one of the chief bases of appeal on the part of the hospitals, and free relief is practically dictated by the public themselves. It is argued that an institution which takes payment does not require charity. The disparity between cost and payment is willingly forgotten, and the encouragement of self-respect and self-reliance appeals little to the sentimental and thoughtless.

